# Indigenous yeast can increase the phenolic acid and volatile ester compounds in Petit Manseng wine

**DOI:** 10.3389/fnut.2022.1031594

**Published:** 2022-12-06

**Authors:** Yanyu Wang, Miao Wang, Wenjuan Li, Xinyuan Wang, Weifu Kong, Weidong Huang, Jicheng Zhan, Guangli Xia, Yilin You

**Affiliations:** ^1^Beijing Key Laboratory of Viticulture and Enology, College of Food Science and Nutritional Engineering, China Agricultural University, Beijing, China; ^2^Yantai Research Institute, China Agricultural University, Yantai, Shandong, China; ^3^Yantai Pula Valley Winery Management Co., Ltd., Yantai, Shandong, China; ^4^College of Pharmacy, Binzhou Medical University, Yantai, Shandong, China

**Keywords:** indigenous yeasts, Petit Manseng wine, phenolic acid compounds, volatile ester compounds, sensory characteristics

## Abstract

**Introduction:**

Indigenous yeasts are generally found in grapes, vineyards, and natural environments. Sequential inoculation and fermentation with non-*Saccharomyces cerevisiae* yeast (H30) and *Saccharomyces cerevisiae* (YT13) also improve the flavor of wine.

**Methods:**

This study sequentially inoculated fermented Petit Manseng and natural grape juice with native H30 and YT13 selected from vineyards in Yantai, China.

**Results and discussion:**

The sensory characteristics of Petit Manseng wine were evaluated by detecting the primary organic acids, phenolic acid compounds, and volatile ester compounds. The results showed that the lactic acid content of the natural wine fermented sequentially with H30 and YT13 increased by 490 μg/L compared with the control group, while the ferulic acid content was 1.4 times that of the single-yeast fermentation group. Furthermore, butyrolactone and anthocyanidin propionate were present in the mixed fermentation group, increasing the aroma complexity of Petit Manseng wine and providing high-quality yeast resources that increase the regional characteristics when producing dry white wine.

## Introduction

Of all the yeasts studied, the most attention has been given to *Saccharomyces cerevisiae*, as it is best adapted to survive in these harsh conditions of a wine ferment. In fact, for the majority of untreated wine ferments, *S. cerevisiae* will be the single dominant yeast present at the end of fermentation (1). *Saccharomyces cerevisiae* is the main microorganism responsible for alcohol fermentation, contributing significantly to the alcohol content, taste, and aroma of wine. The major difference between how wine yeasts produce aroma compounds during fermentation stems from the production of enzymes. Yeasts contain genes that encode enzymes that perform important roles in their survival (2). And modern gene sequencing techniques have shown that some non-*Saccharomyces* yeasts encode for a greater amount of extracellular enzymes than *S. cerevisiae* (3). However, Non-*Saccharomyces cerevisiae* produces a large amount of volatile acid and sulfide during fermentation, giving wine a bad smell and adversely impacting its quality. It is considered the cause of pollution and deterioration during wine production (4). In a study of indigenous *Saccharomyces cerevisiae*, it was found that due to the diversity of indigenous *Saccharomyces* yeasts, there is a much wider range of extracellular enzymes produced during a wild fermentation than when inoculating with a monoculture of *S. cerevisiae*, which played a key role in improving wine flavor (5). Therefore, Indigenous Saccharomyces yeasts isolated from grapes can emphasize the specificity of the terroir, and can contribute to an increased market visibility of wine, due to their production of aromatic compounds which are formed during the fermentation, including higher alcohols, esters, terpenes, and volatile thiols (6).

Indigenous *Saccharomyces cerevisiae* is mainly derived from the microbial population on the surfaces of mature grapes. Due to differences in grape varieties, cultivation methods, and geographic locations, indigenous strains diversely affect the grapes from different regions (7). In Europe, winemakers are more inclined to use indigenous yeast strains when producing wine to increase its typicality (8). In Slovenia, indigenous yeast strains selected from Moscato and Welsh Riesling grapes have been shown to increase the content of 3-mercaptoacetate, linalool, geraniol, and 2-phenylethanol in wine and reduce the 3-mercapto-1-hexanol level. Changes in the concentration of these substances provide Sauvignon Blanc wine with a more intense aroma (6). However, although the NI6 indigenous yeast from the Parr region of South Africa contains high-yield 3-mercapto-1-hexanol, it provides Syrah wines with pleasant “jam”, “smoky”, and “spicy” aromas (9).

The essential role of *Saccharomyces cerevisiae* and non-*Saccharomyces cerevisiae* is also reflected in the color and taste of the wine. The study found that *Schizosaccharomyces pombe* and *Meyerozyma guilliermondii* showed a higher ability than the control group to produce hydroxycinnamate decarboxylase significantly increasing the vitisin A and B derivative yield, enhancing the color stability of the wine (10, 11). In addition, certain types of yeast that degrade or produce organic acids during the fermentation process can also play a role in color protection. Previous studies indicated that the anthocyanin concentration in the sequential fermentation group using *Lachancea percherrima* was 8–10% higher than that of the control group (12). The indigenous *Saccharomyces cerevisiae* from France and Northern Italy produced higher concentrations of glycerin, malic acid, and succinic acid during the fermentation process and lower concentrations of ethanol, acetic acid, and acetaldehyde, enhancing the acidity and softness of the wine (13–16). Various regions in China contain abundant yeast resources that require further exploration. Our research group collected 85 non-*Saccharomyces cerevisiae* samples from different regions in China, including Yantai, Huailai, Fangshan, and Shangri-La, for early-stage testing. This study uses *p*-nitrophenyl-β-D-glucoside (pNPG) and the Bradford method to determine the β-glucosidase activity and protein content of the primary strains. The indigenous wild non-*Saccharomyces cerevisiae*, H30, from the Yantai region displays high β-glucosidase activity and is selected based on its specific enzyme activity (17).

## Materials and methods

### Petit Manseng sample

Five-year-old Petit Mensang collected from Beigezhuang Village, Yantai, China. The harvested and selected samples were pressed by bladder press and the extracted grape juice was used for subsequent experiments.

### Yeast strain

Yeast strains were selected from indigenous *Saccharomyces cerevisiae* YT13 and non-*Saccharomyces cerevisiae* H30 that had been isolated from grapes in Penglai, Yantai, China. The EC1118 commercial yeast (French Raman) was used for the control group. Commercial *Saccharomyces cerevisiae* was cultured in a yeast extract peptone dextrose (YPD) medium (yeast extract 10 g/L, peptone 20 g/L, glucose 20 g/L) to obtain the single strain liquid, while the indigenous yeast, which had been frozen at −80°C, was thawed and rewarmed, then inoculated in YPD liquid medium, and amplified to obtain the single bacterial solution. This single bacterial solution was added into sterile grape juice at the inoculation ratio of 10% (v/v), until the strain grew to more than 10^7^ CFU/mL.

### Chemicals

Anhydrous sodium carbonate, Folin phenol, sodium chloride, acetic acid, methanol, 2-octanol, tartaric acid, malic acid, lactic acid, citric acid and succinate were purchased from Shanghai Macklin Biomedical Co., Ltd. (Shanghai, China). Gallic acid, *p*-coumaric acid, protocatechuic acid, gentian acid, chlorogenic acid, ferulic acid, caffeic acid and vanillic acid were purchased from Shanghai Aladdin Biochemical Technology Co., Ltd. (Shanghai, China). Glucose, agar, and glycerol purchased from China Sinopharm Chemical Reagent Factory (Shanghai, China). Peptone and yeast extract were purchased from Beijing Shuangxuan Microbial Culture Product Medium Factory (Beijing, China). WLN medium was purchased from Shanghai Junrui Biotechnology Co., Ltd. (Shanghai, China). Cross-linked polyvinylpyrrolidone (PVPP) was purchased from Beijing Solarbio Science and Technology Co., Ltd. (Beijing, China). Ethyl acetate was purchased from Tianjin Fuyu Fine Chemical Co., Ltd. (Tianjin, China). Anhydrous ethanol was purchased from Shanghai Forneeds Biotechnology Factory (Shanghai, China). Potassium sulfite was purchased from Enartis (Italy). Pure water was obtained from an Elix ultrapure water purification system.

### Fermentation process

The clarified grape juice was filtered through 1 and 0.45 μm filter membranes to obtain the sterilized grape juice, of which 2 L was transferred into a 2.5 L glass fermentation tank. The control group of this experiment was commercial Saccharomyces cerevisiae EC1118 single strain inoculation and fermentation, and indigenous *Saccharomyces cerevisiae* YT13 was used as another single strain fermentation group. In the mixed strain group, non-*Saccharomyces cerevisiae* H30 was inoculated first, and EC1118 and YT13 were inoculated on the fourth day of fermentation. All inoculate proportion was 1%. After inoculation, all samples were fermented at 16°C. The inoculation grouping and fermentation of the natural grape juice were consistent with the abovementioned steps for the sterilization of grape juice, and the specific grouping is shown in Table 1. The fermentation of the samples was monitored by measuring Brix values daily. At the end of fermentation, residual sugar content in the different treatment groups was detected using a wine analyzer (F17-WineScan FT120, Foss Co., Ltd., Hillerød, Denmark). When sugar content reached below 4 g/L, the fermentation was stopped by the addition of potassium sulfite. The wine was then poured into glass bottles and stored at 4°C for subsequent detection.

**TABLE 1:** Fermentation test groups.

Grape juice nature	Yeast inoculation method	Types of yeast inoculation
Sterilization	Single yeast inoculation	EC1118 (control)
		YT13
	Sequential inoculation	H30 + EC1118
		H30 + YT13
Nature	Single yeast inoculation	EC1118 (control)
		YT13
	Sequential inoculation	H30 + EC1118
		H30 + YT13

This test was performed on two fermentation systems: sterilization and natural fermentation. Each system comprised four groups. Except for the control group (EC1118), the test groups were as follows: YT13 *Saccharomyces cerevisiae* fermentation (YT13); H30 fermentation for 3 days, followed by inoculation with EC1118 for sequential fermentation (H30 + EC1118); H30 fermentation for 3 days, followed by inoculation with YT13 for sequential fermentation (H30 + YT13).

### Determination of organic acids

The organic acids produced by fermentation were detected by high performance liquid chromatography (HPLC), according to a method previously described in the literature. The wine samples were filtered by 0.22 μm aqueous microporous membrane and then analyzed by HPLC. Chromatographic conditions were as follows: Waters XBridge C18 chromatographic column (4.6 mm × 250 mm, 5 μm), column temperature of 35°C, detection wavelength of photodiode array detector (PDA) of 210 nm, isocratic elution, mobile phase ratio of 0.1% phosphoric acid solution:methanol = 97.5:2.5, flow rate of 0.8 mL/min, and injection volume of 10 μL. The establishment of the standard working curve was performed as follows: Five mixed standard samples of different concentrations were accurately prepared, stored at 4°C, and subsequently detected under the above chromatographic conditions. Five standard curves were obtained, with peak area as the ordinate and mass concentration of substance as the abscissa. The peak sequence of each standard sample was found to be as follows: tartaric acid, 3.5 min; malic acid, 4.2 min; lactic acid, 5.18 min; citric acid, 6.55 min; and succinic acid, 7.7 min.

### Determination of phenolic acids

A wine sample of 10 mL was centrifuged after vortex oscillation with ethyl acetate of equal volume. The supernatant was centrifuged three times (10 min each time) in a 100 mL round-bottom flask and then dried by vacuum distillation at 35°C. The residue was dissolved with 2 mL 50% chromatographic methanol and stored at −20°C for subsequent chromatographic analysis (18). The chromatographic conditions were as follows, according to a method slightly modified by Chen (19). For gradient elution, mobile phase A was methanol-acetic acid-water (volume ratio 10:2:88), mobile phase B was methanol, the flow rate was 0.8 mL/min, and in the elution program: 0–25 min, B was 0–15%; 25–45 min, B was 15–50%; and 45–53 min, B was 50–0%. The column temperature was 30°C, injection 20 μL. Standard working solution: 2 mg/mL standard solution was diluted with methanol to 0.05, 5, 15, 30, 40, and 60 mg/L standard solution, then stored at −20°C. Mixed standard working solution: 80 μL of vanillic acid (2 mg/mL) and 100 μL of other phenolic compounds were dissolved together in chromatographic methanol and diluted to 10 mL, which was then used as the mother solution for further use. The solution was diluted into five standard solutions at different concentrations and stored at −20°C until needed. The peak sequences of each standard sample were as follows: gallic acid, 4.3 min; protocatechuic acid, 7.2 min; gentiopic acid, 11.1 min; chlorogenic acid, 12.9 min; caffeic acid, 15.2 min; vanillic acid, 20.1 min; p-coumaric acid, 25.7 min; ferulic acid, 28.65 min. In the qualitative analysis, cholic acid was detected at 320 nm and the other substances were detected at 280 nm. The identification was carried out by comparing the retention time and spectrum with the pure standard. An external method was used for quantitative analysis.

### Determination of volatile esters

Headspace solid phase microextraction gas chromatography-mass spectrometry (HS-*SPME*-*GC-MS)* was used to detect the aroma compounds in samples, as described by Li et al. (20). A 3 mL wine sample was placed in a 10 mL headspace bottle, to which 0.5 g NaCl powder, 0.3 g PVPP, 3 μL concentration of 0.822 g/L 2-octanol internal standard and a magnetic rotor were added, after which it was sealed with a bottle cap with a rubber gasket. The extraction head of the SPME fiber assembly was aged for 1 h at the inlet of the gas chromatography, at an aging temperature of 250°C. The aged extraction head [divinylbenzene/carboxen/*polydimethylsiloxane* (DVB/CAR/PDMS)] was inserted into the headspace part of the sample bottle, 1 cm away from the liquid surface. The sample was heated and stirred at 50°C and 350 rpm for 50 min, whereafter the extraction head was taken out and inserted into the GC inlet (Thermo Scientific) at 250°C for 5 min. Each sample was measured three times in parallel.

The GC conditions were as follows: The column was a TG-5SILMS (30 m × 0.25 mm × 0.25 μm) capillary column. The carrier gas was high purity helium, and the flow rate was 1.2 mL/min. Solid-phase microextraction was manually injected using the non-split mode. The inlet temperature was 250°C. The column temperature heating program was initialized at 40°C, maintained for 2 min, heated to 230°C at the rate of 5°C/min, then maintained for 5 min. The MS conditions were as follows: interface temperature was 250°C, ion source temperature was 230°C, ionization mode was EI, electron ionization energy was 70 eV, full scan mode acquisition signal, and the scanning range (m/z) was 30–450 amu. For the qualitative analysis, different flavor components were separated by GC to form their respective chromatographic peaks, which were analyzed and identified by GC-MS-computer. The mass spectra of each component were retrieved and analyzed via the online National Institute of Standards and Technology (NIST) mass spectral library. Combined with the relevant literature, artificial analysis was carried out to confirm the chemical components of the flavor substances. Only substances with similarity (SI) greater than 600 and triplicate detection were retained. For the semi-quantitative analysis, the following internal standard method was applied: Using an MS full ion scanning (Scan) map, combined with the comparative results of NIST2011 and the related literature reference, the aroma substances were qualitatively screened. Using the semi-quantitative method, the average value of three replicates was taken as the relative content of the aroma substances.

### Sensory evaluation

A tasting group comprised of seven members conducted sensory evaluation and analysis on the dry white wines fermented by different yeasts in this study. A five-point intensity method was used to evaluate the aromas of the fermented products. Seven aroma characteristics, namely alcoholic odor, floral aroma, citrus fruit, tropical fruit, temperate fruit, fermented aroma, and adverse aroma, were selected according to the international Wine and Spirit Education Trust (WSET) wine tasting guidelines. In addition, the appearance (color and clarity), aroma (intensity, coordination, freshness, and complexity) and taste (mellowness, balance, and aftertaste) of each sample were scored, for a total score of 100 points.

### Statistical analysis method

IBM SPSS Statistics 26 software was used for significance difference analysis. Analysis of variance (ANOVA) was used; *n* = 3; and *p* < 0.05 indicated significant difference. The results were expressed as mean ± standard deviation. GraphPad Prism 8.0 software was used to draw the broken line diagrams, scatter plots and column diagrams of chemical components in the samples at different fermentation times. The principal component analysis (PCA) chart was created using OriginPro2021 software, while the flavor radar chart was drawn using Excel software.

## Results

### The effect of indigenous yeast fermentation on the basic physicochemical indexes of Petit Manseng dry white wine

In order to explore the fermentation performance of the two indigenous yeasts (YT13 and H30) in sterilized grape juice and natural grape juice, YT13, H30 + YT13, and H30 + EC1118 were used to ferment Petit Manseng grape juice, while commercial *Saccharomyces cerevisiae* EC1118 was used as the control. The pH value, sugar content, total acid content, alcohol content, and total phenol content of the samples were determined at the beginning and end of fermentation. The results are shown in Table 2.

**TABLE 2:** Basic physical and chemical indicators of Petit Manseng grape juice and wine.

Detection indicator		Sterilization group				Natural group			
	Grape juice	Control	YT13	H30 + EC1118	H30 + YT13	Control	YT13	H30 + EC1118	H30 + YT13
Fermentation days (days)	0	19	22	23	23	13	17	15	15
pH	3.1 ± 0.02	3.1 ± 0.05^a^	3.2 ± 0.02^a^	3.1 ± 0.05^a^	3.2 ± 0.04^a^	3.1 ± 0.04^a^	3.2 ± 0.01^a^	3.1 ± 0.03^a^	3.1 ± 0.03^a^
Sugar content (g/L)	230 ± 0.10	1.9 ± 0.02^a^	2.3 ± 0.01^a^	1.9 ± 0.07^a^	2.0 ± 0.13^a^	1.4 ± 0.01^d^	1.7 ± 0.09^c^	1.9 ± 0.12^b^	2.3 ± 0.05^a^
Total acid (g/L)	8.8 ± 0.04	9.4 ± 0.05^ab^	9.6 ± 0.04^a^	9.3 ± 0.05^b^	9.3 ± 0.04^b^	9.3 ± 0.03^a^	9.3 ± 0.11^a^	9.3 ± 0.10^a^	9.3 ± 0.08^a^
Alcohol (%Vol)	N.D.	13.2 ± 0.06^a^	13 ± 0.10^a^	13.2 ± 0.06^a^	13.1 ± 0.09^a^	13.1 ± 0.07^a^	13 ± 0.10^a^	13.3 ± 0.12^a^	13.2 ± 0.01^a^
Total phenol (mg/L)	N.D.	375.47 ± 10.67^b^	372.17 ± 8.00^b^	394.81 ± 1.33^a^	387.74 ± 2.00a^b^	340.33 ± 7.67^b^	362.97 ± 11.01^a^	354.24 ± 7.34a^b^	295.99 ± 1.00^c^

The control group was inoculated with commercial *Saccharomyces cerevisiae* EC1118 (EC1118), while the test groups are treated as follows: YT13 *Saccharomyces cerevisiae* fermentation (YT13); H30 fermentation for 3 days, followed by inoculation with EC1118 for sequential fermentation (H30 + EC1118); H30 fermentation for 3 days, followed by inoculation with YT13 for sequential fermentation (H30 + YT13). In the table, N.D. means not detected. Different letters indicate significant differences at *p* < 0.05.

The Petit Manseng grape juice displayed an initial pH value of 3.1, an initial sugar content of 230 g/L, and a total acid content of 8.8 g/L. In the sterile group, the sugar content of the wine fermented with commercial *Saccharomyces cerevisiae* EC1118 decreased rapidly, while the fermentation process was completed after 19 days. The indigenous *Saccharomyces cerevisiae* YT13 obtained from Penglai was used in the single-yeast fermentation and sequential inoculation fermentation groups, while the fermentation process was completed 23 days after yeast inoculation. The fermentation speed of the Petit Manseng natural grape juice was faster than the fermentation process of the sterilized grape juice, which could be attributed to the loss of macromolecular nutrients during filtration and sterilization. The viability and sugar consumption rate of the commercial *Saccharomyces cerevisiae* EC1118 (control group) was higher in both groups, reducing the fermentation process. After fermentation, the pH of the Petit Manseng wine displayed minimal changes, while the total acid content was significantly higher than that of the grape juice. This could be ascribed to the effect of the yeast, which increased the organic acid content, such as lactic acid and succinic acid. Previous studies showed that different yeast strains affected the polyphenol content in wine. In this study, the result regarding the total phenol content in the Petit Manseng wine fermented with different yeasts showed no significant differences between the sterilization groups. The total phenol content in the natural grape juice group fermented with *Saccharomyces cerevisiae* YT13 was 362.97 ± 11.01 mg/L, which was significantly higher than in the control group. However, the total phenol content of the mixed fermentation group using H30 + YT13 was significantly lower than in all treatment groups.

### Indigenous yeast increases the organic acid content of Petit Manseng dry white wine

As shown in Table 3, the type, concentration, and ratio of organic acids play a vital role in wine and can adjust the acid-base balance, maintain the pH value at a relatively low level, affect the taste, and even improve the aroma by affecting the esterification reaction. Organic acids are not only essential for the taste of wine but also determine its acidity. Studies have shown that the content and ratio of organic acids produced by different yeast strains vary. Therefore, this study used liquid chromatography to examine the wine fermented using grape juice and natural grape juice. Five organic acids were detected, including tartaric acid, malic acid, succinic acid, lactic acid, and lemon acid.

**TABLE 3:** The content of organic acids in wine fermented from Petit Manseng grape juice.

Organic acid content (g/L)	Sterilization group				Natural group			
	Control	YT13	H30 + EC1118	H30 + YT13	Control	YT13	H30 + EC1118	H30 + YT13
Tartaric acid	4.05 ± 0.34^ab^	3.79 ± 0.03^b^	3.92 ± 0.01^ab^	4.39 ± 0.03^a^	4.11 ± 0.09^ab^	3.97 ± 0.03^bc^	3.84 ± 0.01^c^	4.20 ± 0.01^a^
Malic acid	6.25 ± 0.30^a^	4.10 ± 3.34^a^	6.66 ± 0.40^a^	6.96 ± 0.43^a^	6.71 ± 0.08^b^	6.77 ± 0.03^a^	7.31 ± 0.65^a^	6.96 ± 0.10^a^
Lactic acid	1.24 ± 0.001^c^	1.64 ± 0.01^ab^	1.37 ± 0.25^bc^	1.87 ± 0.10^a^	1.38 ± 0.09^a^	1.61 ± 0.01^a^	1.56 ± 0.01^a^	1.87 ± 0.07^a^
Citric acid	0.67 ± 0.03^a^	0.86 ± 0.27 ^a^	0.51 ± 0.54^a^	0.69 ± 0.01^a^	0.75 ± 0.04^b^	0.79 ± 0.05^b^	1.02 ± 0.01^a^	0.85 ± 0.03^b^
Succinic acid	2.04 ± 0.05^a^	1.99 ± 0.16 ^a^	1.33 ± 0.78^a^	1.14 ± 0.07^a^	0.87 ± 0.03^b^	1.82 ± 0.02^a^	1.66 ± 0.01^a^	0.70 ± 0.19^b^
Total content	14.16 ± 0.40	12.4 ± 2.69	13.80 ± 1.41	15.06 ± 0.41	13.83 ± 0.25	14.96 ± 0.02	15.40 ± 0.48	14.52 ± 0.27

The control group was inoculated with commercial *Saccharomyces cerevisiae* EC1118 (EC1118), while the test groups are treated as follows: YT13 *Saccharomyces cerevisiae* fermentation (YT13); H30 fermentation for 3 days, followed by inoculation with EC1118 for sequential fermentation (H30 + EC1118); H30 fermentation for 3 days, followed by inoculation with YT13 for sequential fermentation (H30 + YT13). Different letters indicate significant differences at *p* < 0.05.

During the fermentation test of the Petit Manseng sterilized grape juice, no significant differences were evident in the malic acid, citric acid, and succinic acid content. However, the lactic acid and tartaric acid levels vary significantly. The lactic acid content (1.87 ± 0.10 g/L) in the H30 + YT13 group was the highest, significantly exceeding the control group level (1.24 ± 0.001 g/L). Although the tartaric acid content difference was not as significant as that of lactic acid, its level in the H30 + YT13 group (4.39 ± 0.03 g/L) was still significantly higher than the control group (4.05 ± 0.34 g/L). Except for the lactic acid content, significant differences were evident in the Petit Manseng wine fermented with natural grape juice. The tartaric acid content (4.20 ± 0.01 g/L) was highest in the wine of the H30 + YT13 group, which was consistent with the results of the sterilization system. However, these results were inconsistent with previous studies in which the malic acid content of cider fermented with the mixed strain containing *Schizosaccharomyces pombe* and *Saccharomyces cerevisiae* was lower than that of the wine subjected to single yeast fermentation. Although the lactic acid content of the three groups, ranging from 1.56 to 1.61 g/L, showed no significant differences, these values were all higher than in the control group (1.38 ± 0.09 g/L). The citric acid content (1.02 ± 0.01 g/L) of the H30 + EC1118 group was significantly higher than in the other groups. The succinic acid test results differed from that of the sterilization system, and its content in the YT13 and H30 + EC1118 groups was significantly higher than in the other two groups. In general, whether it was the sterile or natural group, the organic acid content in wine fermented with indigenous yeast was significantly higher than that in the control group.

### Indigenous yeast increases the phenolic acid content of Petit Manseng dry white wine

Phenolic acids represent important polyphenolic compounds in wine, displaying antioxidant properties and playing a role in human nutrition and health. This study detected a total of eight phenolic acids (namely gallic acid, protocatechuic acid, gentisic acid, chlorogenic acid, caffeic acid, vanillic acid, *p*-Coumaric acid, and ferulic acid) in the wines fermented with filtered and sterilized grape juice.

As shown in Table 4, no significant differences were evident in gallic acid, caffeic acid, vanillic acid, and ferulic acid in the sterilized Petit Manseng grape juice fermented wine. Except for coumaric acid, the phenolic acid content of the single-bacteria and mixed-bacteria fermentations using YT13 was higher than in the control group, confirming the high quality of indigenous strains. However, the results for the H30 + EC1118 mixed fermentation exhibited an opposite trend. The protocatechuic acid (4.24 ± 0.405 g/L), gentisic acid (0.40 ± 0.04 g/L), chlorogenic acid (1.86 ± 0.47 g/L), and soybean acid (0.07 ± 0.09 g/L) levels were all significantly lower than the control group. In the H30 + YT13 mixed fermentation group, the levels of these four phenolic acids were 5.15 ± 0.08, 0.57 ± 0.05, 2.63 ± 0.01, and 0.12 ± 0.04 g/L, significantly higher than the H30 + EC1118 group. It is speculated that this is due to a decrease in the phenolic acid content caused by the interaction between H30 and EC1118. However, whether the significant difference between the phenolic acid production by YT13 and EC1118 is related to the characteristics of the individual yeasts remains unclear and requires further exploration. Furthermore, the indigenous microorganism variety in the Yantai natural grape juice caused changes in the phenolic acid levels. As shown in Table 4, the highest total phenolic acid content in the Petit Manseng wine fermented with natural grape juice reached 16.36 ± 1.9 mg/L, while the levels in the three test groups were higher than in the control group (14.03 ± 0.84 mg/L). Except for gallic acid and caffeic acid, significant differences were apparent in the content of the other phenolic acids. The protocatechuic acid levels in the three groups were 4.42 ± 0.315, 4.49 ± 0.23, and 4.63 ± 0.63 mg/L, which were significantly higher than in the control group (4.08 ± 0.08 mg/L), while the vanillic acid content (0.15 ± 0.004 mg/L) was highest in the YT13 test group. The H30 + EC1118 group displayed the highest gentisic acid level, reaching 0.68 mg/L. The chlorogenic acid detection results were consistent with the sterilization system, exhibiting the highest level (3.43 ± 0.14 mg/L) in the YT13 test group. The *p*-coumaric acid content (0.39 ± 0.05 mg/L) of the H30 + EC1118 group was the highest of the four groups.

**TABLE 4:** The content of phenolic acids in wine fermented from Petit Manseng grape juice.

Phenolic acid content (mg/L)	Sterilization group				Natural group			
	Control	YT13	H30 + EC1118	H30 + YT13	Control	YT13	H30 + EC1118	H30 + YT13
Gallic acid	3.79 ± 0.14^a^	4.32 ± 0.12^a^	4.37 ± 0.16^a^	4.17 ± 0.24^a^	3.85 ± 0.13^a^	4.33 ± 0.42^a^	4.26 ± 0.49^a^	4.29 ± 0.50^a^
Protocatechuic acid	4.42 ± 0.32^b^	5.62 ± 0.21^a^	4.24 ± 0.405^c^	5.15 ± 0.08^a^	4.08 ± 0.08^c^	4.42 ± 0.315^b^	4.49 ± 0.23^b^	4.63 ± 0.63^a^
Gentisic acid	0.46 ± 0.01^ab^	0.59 ± 0.10^a^	0.40 ± 0.04^b^	0.57 ± 0.05^a^	0.53 ± 0.06^b^	0.62 ± 0.16^a^	0.68 ± 0.05^a^	0.55 ± 0.06^b^
Chlorogenic acid	2.29 ± 0.91^ab^	3.50 ± 0.45^a^	1.86 ± 0.47^b^	2.63 ± 0.01^ab^	2.71 ± 0.33^ab^	3.43 ± 0.14^a^	2.59 ± 0.22^b^	3.26 ± 0.38^ab^
Caffeic acid	2.43 ± 0.02^a^	2.43 ± 0.71^a^	2.76 ± 0.04^a^	2.64 ± 0.30^a^	2.01 ± 0.13^a^	2.30 ± 0.19^a^	2.425 ± 0.40^a^	2.45 ± 0.25^a^
Vanillic acid	0.11 ± 0.002^a^	0.14 ± 0.01^a^	0.11 ± 0.01^a^	0.14 ± 0.01^a^	0.11 ± 0.001^b^	0.15 ± 0.004^a^	0.12 ± 0.01^b^	0.12 ± 0.01^b^
P-coumaric acid	0.35 ± 0.14^a^	0.12 ± 0.03^ab^	0.07 ± 0.09^b^	0.12 ± 0.04^ab^	0.34 ± 0.09^a^	0.11 ± 0.12^c^	0.39 ± 0.05^a^	0.29 ± 0.01^b^
Ferulic acid	0.43 ± 0.03^a^	0.55 ± 0.09^a^	0.45 ± 0.02^a^	0.54 ± 0.01^a^	0.41 ± 0.02^d^	0.51 ± 0.06^c^	0.61 ± 0.09^b^	0.78 ± 0.05^a^
Total content	14.27 ± 1.76	17.27 ± 3.63	14.25 ± 3.39	15.95 ± 1.00	14.03 ± 0.84	15.88 ± 2.42	15.58 ± 1.54	16.36 ± 1.9

The control group was inoculated with commercial *Saccharomyces cerevisiae* EC1118 (EC1118), while the test groups are treated as follows: YT13 *Saccharomyces cerevisiae* fermentation (YT13); H30 fermentation for 3 days, followed by inoculation with EC1118 for sequential fermentation (H30 + EC1118); H30 fermentation for 3 days, followed by inoculation with YT13 for sequential fermentation (H30 + YT13). Different letters indicate significant differences at *p* < 0.05.

These results indicated that the levels of other phenolic acid compounds in the YT13-fermented wine were higher than in the control group except for coumaric acid. The total phenolic acid content also verified this conclusion.

### Indigenous yeast increases the relative content of the volatile ester compounds in Petit Manseng dry white wine

**FIGURE 1:**
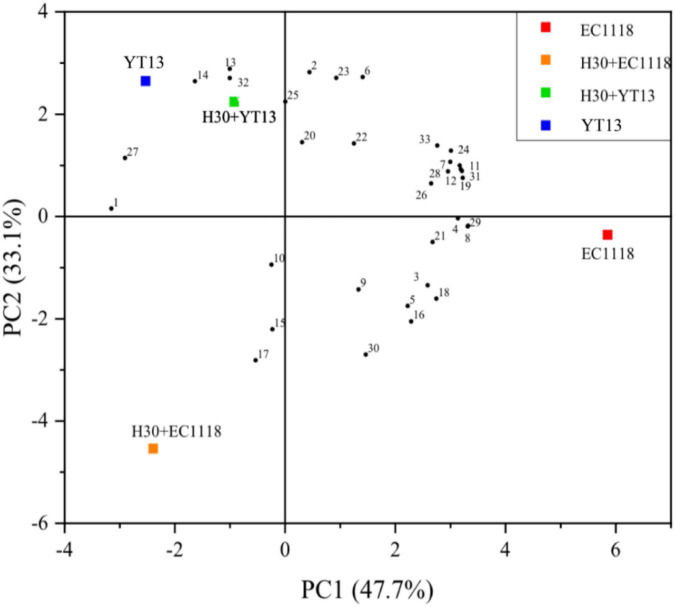
Principal component analysis diagram of the volatile compounds in wine fermented with sterilized Petit Manseng grape juice. The control group was inoculated with commercial *Saccharomyces cerevisiae* EC1118 (EC1118). YT13 refers to inoculation with YT13 *Saccharomyces cerevisiae*. H30 + EC1118 indicates that H30 and EC1118 are inoculated in sequence. H30 + YT13 indicates that H30 and YT13 are inoculated in sequence. PCA analysis is used to select the volatile compounds shared by the different yeast fermentation groups. The black dots with serial numbers in the figure represent volatile compounds: (1) 2-hexyn-1-ol, (2) cis-3-penten-1-ol, (3) 2-octyn-1-ol, (4) nerol, (5) 3,7,11-trimethyldodecanol, (6) 13-heptadene-1-ol, (7) 1-pentadecanol, (8) methyl myristelaidate, (9) 2-hydroxymyristic acid, (10) ethyl 2-non-ynoate, (11) 4-pentenoic acid, (12) 2-hydroxymyristic acid, (13) 9-hexadecenoic acid, ethyl ester, (14) traumatic acid, (15) ethyl phenylacetate, (16) 2-hydroxy-1-methyl propyl ester, (17) ethyl butanoate, (18) phenethyl caproate, (19) dodecyl acrylate, (20) geranyl isovalerate, (21) ethyl caprate, (22) γ-oryzanol, (23) methyl(9Z)-9-hexadecenoate, (24) methyl laurate, (25) methyl 6-octadecenoate, (26) 3-eicosanone, (27) cyclopentadecanone, (28) cyclohexadienone, (29) 1,2-epoxyheptane, (30) coumarin, (31) 3,4-diethylbiphenyl, and (32) 2,6-di-tert-butylhydroquinone, and (33) valerenic acid.

Volatile compounds are affected by many factors. Studies have found that gallic acid and *p*-coumaric acid can spontaneously bind to linalool and its glycosides via hydrogen bonding and dispersion, effectively controlling the release and regulating the overall aroma of wine features. Therefore, this study used gas chromatography and mass spectrometry for volatile compound detection. The sterilized Petit Manseng wine exhibited 55 different volatile compounds, including 18 alcohols, 16 acids, 12 esters, six aldehydes and ketones, and three other compounds. A total of 52 different volatile aroma components were detected in the natural Petit Manseng wine, including 16 alcohols, 16 acids, 12 esters, five aldehydes and ketones, and three other compounds. To more intuitively compare the differences between the natural wines and the sterile wines fermented with different yeasts, the OriginPro 2021 software was used to perform principal component analysis on the data via dimensionality reduction processing. Figure 1 shows the principal component analysis results regarding the volatile compounds in the sterilized Petit Manseng wine. The contribution rates were 47.7% for the first principal component (PC1) and 33.1% for the second principal component (PC2). The cumulative variance contribution rate of the principal components reached 80.8%, while the information loss rate was only 19.2%. The information of the original indicators was mostly retained and used as the principal component to analyze the sterilized Petit Manseng wine. PC1 was mainly composed of alcohol and acid compounds, such as nerolidol and myristic acid, while PC2 primarily consisted of ester compounds providing volatile floral and fruity flavors and included ethyl phenylacetate, ethyl butyrate, isovalerate, and methyl apiate. EC1118 accumulated more aroma compounds, displaying the highest levels in the Petit Manseng wine treated with yeast. The specific types and content of the volatile ester compounds are shown in Table 5. The total relative content of the ester compounds in the YT13 group was 6,686.41 μg/L, which was significantly higher than in the control group. Ethyl phenylacetate, ethyl butyrate, phenethyl caproate, geranyl isovalerate, and ethyl decanoate accounted for a large proportion of the detected ester compounds. The H30 + YT13 group exhibited the most volatile ester compounds (15 types). In addition, the mixed fermentation group contained pearlitol, butyrolactone, and neryl propionate, which were not during single yeast fermentation, increasing the aroma complexity of the Petit Manseng wine exposed to the mixed fermentation process.

**TABLE 5:** Relative content of volatile ester compounds in wine fermented from Petit Manseng grape juice.

Relative content (μg/L)	Sterilization group				Natural group			
	Control	YT13	H30 + EC1118	H30 + YT13	Control	YT13	H30 + EC1118	H30 + YT13
Ethyl cinnamate	N.D.	212.08 ± 2.08^b^	20.46 ± 1.30^c^	241.78 ± 13.47^a^	N.D.	214.23 ± 0.23^a^	175.78 ± 7.39^b^	174.69 ± 13.40^b^
Ethyl phenylacetate	238.54 ± 73.72^ab^	157.19 ± 38.28^b^	255.87 ± 68.59^ab^	304.67 ± 12.24^a^	402.25 ± 18.88^a^	284.58 ± 0.36^b^	308.01 ± 17.84^ab^	394.34 ± 94.26^a^
Ethyl caproate	40.33 ± 5.14^a^	72.40 ± 35.58^a^	N.D.	N.D.	86.16 ± 6.47^a^	68.39 ± 9.43^b^	N.D.	N.D.
Ethyl butyrate	2317.58 ± 215.99^a^	2518.64 ± 231.79^a^	2026.92 ± 218.02^a^	2736.74 ± 580.95^a^	3028.41 ± 360.42^a^	2491.05 ± 40.00^b^	3138.06 ± 91.35^a^	3235.4 ± 83.98^a^
Phenethyl caproate	117.90 ± 24.19^b^	121.89 ± 19.65^ab^	142.35 ± 8.49a^b^	155.51 ± 14.99^a^	333.35 ± 62.54^a^	240.8 ± 29.57^b^	310.09 ± 14.62^ab^	358.05 ± 3.57^a^
Geranyl isovalerate	1650.23 ± 79.97^bc^	2257.39 ± 189.69^a^	1290.54 ± 130.07^c^	1984.11 ± 330.95^ab^	3291.6 ± 421.80^b^	2590.96 ± 127.07^c^	3733.87 ± 128.14^a^	3832.07 ± 77.43^a^
Ethyl decanoate	13.88 ± 1.52^ab^	15.65 ± 0.53^a^	11.76 ± 1.70^b^	15.76 ± 0.99^a^	21.19 ± 0.16^a^	20.45 ± 5.26^a^	23.26 ± 0.27^a^	24.77 ± 6.26^a^
Methyl decanoate	123.25 ± 16.97^b^	132.68 ± 13.83^b^	161.02 ± 32.15^b^	305.24 ± 20.87^a^	73.85 ± 13.58a^b^	56.85 ± 2.56^b^	64.66 ± 0.20^ab^	84.62 ± 17.85^a^
Ethyl linoleate	24.50 ± 4.73^b^	30.96 ± 3.40^a^	11.59 ± 0.81^c^	27.56 ± 1.02^ab^	39.58 ± 0.25^a^	38.31 ± 7.15^a^	38.08 ± 0.06^a^	40.47 ± 20.73^a^
Ferulate	22.64 ± 1.90^b^	30.88 ± 3.63^a^	18.21 ± 7.11^b^	19.75 ± 0.81^b^	23.51 ± 2.60^ab^	23.37 ± 2.90^ab^	26.55 ± 0.97^a^	21.17 ± 2.81^b^
Hexyl acetate	14.62 ± 2.60^ab^	18.33 ± 1.80^ab^	20.86 ± 5.17^a^	14.13 ± 0.13^b^	22.26 ± 1.48^a^	22.17 ± 3.94^a^	21.88 ± 0.19^a^	23.3 ± 9.31^a^
Methyl laurate	26.78 ± 15.45^a^	34.40 ± 25.10^a^	18.43 ± 10.68^a^	11.31 ± 4.62^a^	35.49 ± 7.04^bc^	18.39 ± 11.67^c^	44.27 ± 13.08^b^	100.43 ± 15.89^a^
Total esters	4,590.25	5,602.49	3,978.01	5,815.78	7,357.65	6,069.55	7,884.51	8,289.31

The control group was inoculated with commercial *Saccharomyces cerevisiae* EC1118 (EC1118), while the test groups are treated as follows: YT13 *Saccharomyces cerevisiae* fermentation (YT13); H30 fermentation for 3 days, followed by inoculation with EC1118 for sequential fermentation (H30 + EC1118); H30 fermentation for 3 days, followed by inoculation with YT13 for sequential fermentation (H30 + YT13). In the table, N.D. means not detected. Different letters indicate significant differences at *p* < 0.05.

**FIGURE 2:**
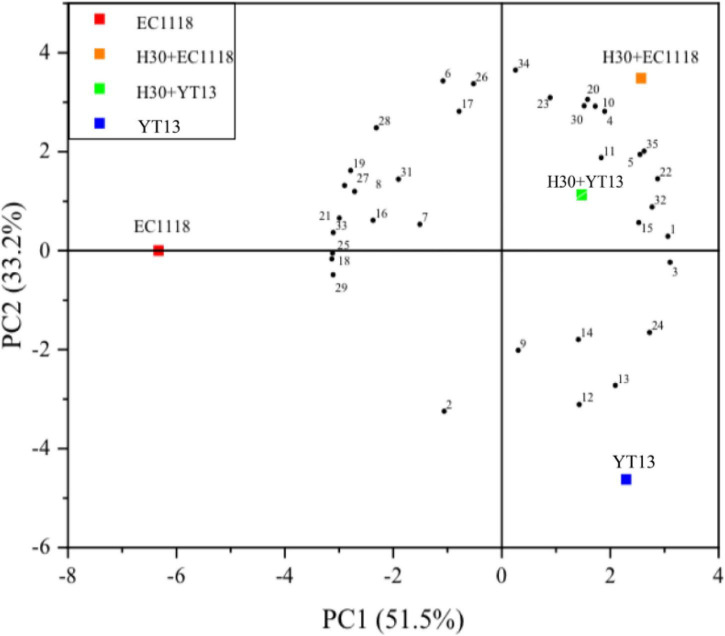
Principal component analysis diagram of the volatile compounds in wine fermented with Petit Manseng natural grape juice. The control group was inoculated with commercial *Saccharomyces cerevisiae* EC1118 (EC1118). YT13 refers to inoculation with YT13 *Saccharomyces cerevisiae*. H30 + EC1118 indicates that H30 and EC1118 are inoculated in sequence. H30 + YT13 indicates that H30 and YT13 are inoculated in sequence. PCA analysis is used to select the volatile compounds shared by the different yeast fermentation groups. The black dots with serial numbers in the figure represent volatile compounds: (1) 4-methylpentan-2-ol, (2) 2,4,5-trimethylbenzaldehyde, (3) cis-3-penten-1-ol, (4) cis-4-cyclopentene-1,3-diol, (5) nerolidol, (6) 2-octyn-1-ol, (7) linalool, (8) nerol, (9) 1-pentadecanol, (10) myristic acid, (11) 2-hydroxymyristic acid, (12) 2-decanoic acid, (13) 4-pentenoic acid, (14) 2-hydroxymyristic acid, (15)9-hexadecenoic acid,(16) traumatic acid, (17) ethyl phenylacetate, (18) 2-hydroxy-1-methyl propyl ester, (19) ethyl butanoate, (20) phenethyl caproate, (21) dodecyl acrylate, (22) geranyl isovalerate, (23) ethyl caprate, (24) γ-oryzanol, (25) methyl vaccenate, (26) methyl (9Z)-9-hexadecenoate, (27) methyl laurate, (28) neryl acetate, (29) methyl 6-octadecenoate (30) 5-Octadecanone, (31) 3-eicosanone, (32) cyclohexadienone, (33) 1,2-epoxyheptane, (34) coumarin, and (35) 3,4-diethylbiphenyl.

The interaction between the grape juice and other microorganisms in the environment and native yeast remains unclear, while the metabolite production may also affect the volatile compounds. Figure 2 shows the principal component analysis results for the volatile compounds in natural Petit Manseng wine. The contribution rates were 51.5% for PC1 is 51.5 and 33.2% for PC2. The cumulative variance contribution rate of the two principal components reached 84.7%, mostly retaining the information of the original indicators. Therefore, these two principal components can be used for two-dimensional plane analysis. In the Figure 2, YT13 was distributed in the third quadrant, while the H30 + EC1118 and H30 + YT13 mixed fermentation groups were distributed in the second quadrant. Furthermore, various aroma compounds were clustered together, including myristic acid, phenethyl caproate, and ethyl caprate. These findings indicated that the mixed fermentation group was richer in aroma types and content and established a good aroma base. Furthermore, the results showed that the total relative content and types of ester compounds in the H30 + EC1118 group were the highest, yielding a total content value of 9,370.99 μg/L for 16 compounds, followed by the H30 + YT13 test group. In addition, similar to the sterilization system, both pearlitol and butyrolactone were detected in the mixed fermentation group, increasing the complexity of the volatile aroma.

### Indigenous yeast enhances the fruity and floral characteristics of Petit Manseng dry white wine

**FIGURE 3:**
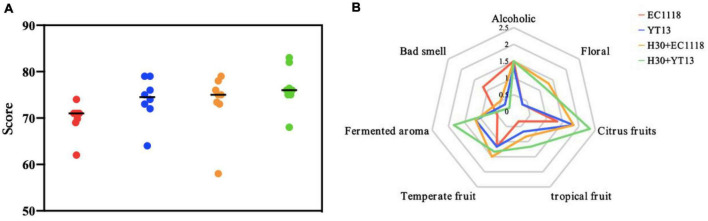
Sensory scoring map **(A)** and aroma evaluation radar map **(B)** of wine fermented with Petit Manseng sterilized grape juice. The control group was inoculated with commercial *Saccharomyces cerevisiae* EC1118 (EC1118); YT13 indicates inoculation with YT13 *Saccharomyces cerevisiae*; H30 + EC1118 indicates sequential inoculation with H30 and EC1118; H30 + YT13 indicates sequential inoculation with H30 and YT13.

Instrumental analysis and sensory appraisal were combined to evaluate the influence of different strains on the aroma of the dry white wine more comprehensively. Based on Gas Chromatography-Mass Spectrometer (GC-MS) instrumental analysis, seven professionals with extensive experience in wine tasting were invited to perform a sensory evaluation on the fermented Petit Manseng dry white wine, assessing the appearance (color and clarity), aroma (richness, coordination, freshness, and complexity), and taste (body, balance, and aftertaste). A total of 100 points was scored. As shown in Figure 3A, the H30 + YT13 group of sterilized Petit Manseng wine received the highest average sensory score of 76.4 points, which could be attributed to a lower content of higher alcohols, while the ester compounds displayed the most variety but little content, maintaining the balance and coordination of the wine. This was followed by the YT13 group with 74 points and the H30 + EC1118 treatment group with 73.4 points. However, the control group received the lowest score due to higher volatile acid content, displaying a boiled smell and sour taste. In addition, pearlitol, butyrolactone, and neryl propionate were only detected in the mixed fermentation group, providing enhanced floral, fruity, and fermented aromas. Therefore, the wines fermented with mixed bacteria received higher aroma scores.

Figure 3B shows the flavor radar chart of the smell evaluation of the sterilized Petit Manseng wine, displaying descriptive groups, such as “alcohol smell,” “floral fragrance,” “citrus fruits,” “temperate fruits,” and “fermented aroma.” A significant difference was evident in attribute strength. The H30 + EC1118 group displayed the highest floral aroma (orange blossom) and temperate fruit aroma (peach and apricot), which was related to the high concentration of ethyl butyrate. Significant differences were apparent in the ferulate, palmitoleate, and neryl propionate levels of the H30 + YT13 group. Therefore, this group received the highest aroma evaluation score regarding citrus fruit (citrus and kumquat), tropical fruit (melon and pineapple), and fermented aromas (bread). Except for the undesirable and floral aromas, the other aroma scores of the control group were the lowest.

**FIGURE 4:**
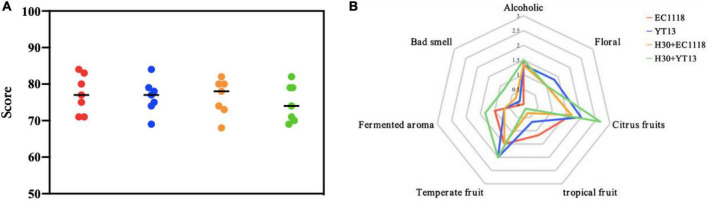
Sensory scoring map **(A)** and aroma evaluation radar map **(B)** of wine fermented with Petit Manseng natural grape juice. The control group was inoculated with commercial *Saccharomyces cerevisiae* EC1118 (EC1118); YT13 indicates inoculation with YT13 *Saccharomyces cerevisiae*; H30 + EC1118 indicates sequential inoculation with H30 and EC1118; H30 + YT13 indicates sequential inoculation with H30 and YT13.

The natural wine was scored a total of 100 points according to appearance, aroma, and taste. The scores are shown in Figure 4A. The control group received the highest average score of 77.3 points, followed by the YT13 group with 76.6 points and the H30 + EC1118 group with 76.4 points. Since the YT13 group may contain higher decanoic acid levels, an undesirable fatty smell and abrasive taste may affect the final sensory score. Figure 4B shows the radar chart of the smell and flavor evaluations of the wine fermented with natural Petit Manseng grape juice. Due to its specific characteristics, the citrus fruit (lemon, citrus, and kumquat) and temperate fruit (apricot and peach) aromas provided by the Petit Manseng grape variety generally scored higher than other flavor properties. The YT13 group displayed the highest floral fragrance (neroli and honeysuckle), while the total alcohol and acid compound content was prominent. The YT13 group obtained the highest floral fragrance score due to the compound fragrance of higher alcohols. Since 2-hydroxymyristic acid and linalool showed significant differences, more intense sweet orange and cool lemon aromas were evident in the H30 + YT13 group.

## Discussion

This study explored two indigenous yeast strains from Yantai. The results showed that the indigenous *Saccharomyces cerevisiae* YT13 displayed excellent fermentation performance, significantly increasing the gallic acid, protocatechuic acid, gentisic acid, and caffeic acid content in Petit Manseng wine and reducing the coumaric acid concentration. The lactic acid content, which most substantially improved the taste, also increased significantly. The ferulic acid content in the sequential fermentation group using the indigenous non-*Saccharomyces cerevisiae*, H30, was 1.4 times higher during mixed fermentation than single yeast fermentation. In addition, due to the different metabolic pathways of different yeasts, obvious differences were evident between the types of trace aroma compounds and their respective contents (21). Pearlitol, butyrolactone, and neryl propionate were detected in the wine of the mixed fermentation group. These trace volatile compounds provided creamy, orange blossom, melon, and pineapple aromas.

The sharp acidity of a wine is reduced by malolactic fermentation (MLF). It is speculated that there may be a positive symbiotic relationship between lactic acid bacteria and indigenous yeast, which is also reflected in this research (22). The lactic acid content is used as a vital reference index to judge the taste of wine. The experiment revealed that the lactic acid content increased significantly during the alcohol fermentation stage when indigenous yeasts participated in fermentation. This phenomenon is also reflected in the mixed yeast fermentation, suggesting that it may be related to the non-*Saccharomyces cerevisiae* participating in the synergistic yeast interaction. The advantage of mixed fermentation is also reflected in the aroma of the wine. A study involving Syrah wine indicated that the interaction between *Oenococcus*, *Lactobacillus plantarum*, and yeast affected the mannoprotein content. Furthermore, the ethyl lactate level also increased significantly, enhancing the aroma of the wine (23–25).

Although the positive effect of phenolic acid compounds on the human body is currently attracting considerable research attention, minimal studies are available regarding the high-yield phenolic acid compounds in indigenous yeast. The indigenous yeast used in this study increased the levels of phenolic acid compounds, such as protocatechuic acid, gallic acid, and caffeic acid via fermentation. This is more pronounced during mixed fermentation and may be attributed to the interaction between the yeasts or between the yeast and phenolic acid (26). The metabolism in the digestive tract and the interaction between the intestinal flora and polyphenolic compounds, such as para-hydroxycinnamic acid, ferulic acid, *p*-Coumaric acid, and caffeic acid, have been proven effective against non-alcoholic fatty liver disease and obesity (27). In addition, the influence of phenolic compounds on the color strength and stability of wine has attracted significant attention. For example, caffeic acid can increase the color saturation of wine, while the chromaticity characteristics and phenolic composition of wine with exogenous ellagic acid are superior to wine with gallic acid (28, 29).

Regarding volatile ester compounds, pronounced differences were evident between the types of aroma compounds and their respective content due to the different metabolic yeast pathways (30). Previous research screened the indigenous *Hanseniaspora uvarum* H30 strain that caused the wine to release more terpene aroma substances. The H30 yeast displayed higher β-glucosidase activity, catalyzing glycosidic bond hydrolysis in the aroma substance molecule in a glycoside-bound state. Studies have shown that adding β-glucosidase BGL0224 during the fermentation process can increase the “tropical fruit” and “floral” scents of Cabernet Sauvignon (17, 31). However, the interaction between *Saccharomyces cerevisiae* and non-*Saccharomyces cerevisiae* may also lower the β-glucosidase activity, significantly reducing the nerol produced by β-glucosidase hydrolysis during mixed fermentation. This study detected no isoamyl lactate in the wine of the sterilization system, indicating to a certain extent that other microorganisms in the grape juice interact with *Saccharomyces cerevisiae*, modifying the aroma compound content (32). The competition for nitrogen source nutrients directly affects the fermentation performance of yeast. Due to the different metabolic yeast pathways to nitrogen sources, a two-pronged strategy using isotope labeling and RNA sequencing has been explored. Moreover, the metabolic network of nitrogen utilization in *Kluyveromyces marxianus* and its regulatory mechanism has been analyzed, showing the ultimate effect of the aroma compound content on key wine varieties (33–35). *Saccharomyces cerevisiae* can also act on the phenolic acid and norisoprene aroma precursors in grapes via specific enzyme activity to effectively regulate the aroma of the variety and reduce the odor during the aging process (36).

Furthermore, differences were evident in the effect of indigenous yeast on the two different systems of the sterile and natural groups. During the fermentation process of the sterilized grape juice, the *Saccharomyces cerevisiae* and non-*Saccharomyces cerevisiae* used in the experiment displayed a competitive reproduction advantage, reflecting the influence of indigenous yeast on the quality of dry white wine. However, a large number of microorganisms are introduced into the environment during actual natural wine production. The interaction between these unknown microorganisms and the yeast may affect the metabolic pathways and enzyme activity. The difference between the two systems is particularly obvious during the fermentation process. Since the natural grape juice only required a short fermentation time, the fermentation rate was significantly higher than sterilized grape juice. The most important reason for this is that grape juice is a natural nutrient containing abundant microbial resources, such as indigenous yeast, lactic acid bacteria, acetic acid bacteria, hop bacteria, *Bacillus bitterus*, and molds. In addition to indigenous yeasts that provide wine with its unique flavor, hop bacteria can produce a strange acetaldehyde odor. Acetic acid bacteria produce a gray film on the surface of the wine during the fermentation process, causing a bad acetic acid taste (24, 37, 38). The phenolic acid results showed that the protocatechin and gentisic acid levels in the natural wine group were slightly higher than in the sterile wine group. The findings regarding the volatile aroma compounds indicated that the relative ester content in the wine fermented from natural grape juice was 2.3 times higher than wine fermented from sterile grape juice. However, the reasons and mechanisms regarding these effects required further exploration. In addition, whether a macromolecular nutrient loss occurs during the sterilization operation necessitates verification.

Water, soil, and terroir have cultivated relatively fixed microbial populations over an extended period. The characteristics of wine-producing areas are affected by the local climate, soil, and raw grape materials, as well as indigenous microorganisms, including *Saccharomyces cerevisiae*, non-*Saccharomyces cerevisiae*, and lactic acid bacteria (39, 40). For example, the Pinot Noir wines from various production areas in Australia are affected by the terroir, resulting in certain differences in mineral content (41). Furthermore, wine from different production areas shows unique regional sensory characteristics. “Mint” and “dark fruit” are important qualities of Coonawarra wines, while wine from the Margaret River region exhibit typical “floral” and “green pepper” flavors (42). In addition, a study involving Burgundy confirmed the uniqueness of the village-level production area (40). The microorganisms in different areas interact with the raw grape materials to produce metabolites that vary in type and content, including organic acids, phenolic acids, volatile compounds, and other products, affecting the sensory quality and characteristics of wines worldwide. Therefore, the impact of terroir and microorganisms requires further exploration.

## Conclusion

The indigenous *Saccharomyces cerevisiae* YT13 can significantly enhance the lactic acid content in Petit Manseng dry white wine, increase the protocatechuic acid, caffeic acid, gallic acid, and ferulic acid concentrations in the phenolic acid, and promote the production of volatile ester compounds. Sequential inoculation and fermentation with indigenous non-*Saccharomyces cerevisiae* H30 and indigenous *Saccharomyces cerevisiae* YT13 can improve the quality of Petit Manseng dry white wine and promote the embodiment of the regional characteristics.

## Data availability statement

The original contributions presented in this study are included in the article/supplementary material, further inquiries can be directed to the corresponding authors.

## Ethics statement

The studies involving human participants were reviewed and approved by China Agricultural University, Tsinghua East Road 17, Haidian District, Beijing, 100083, China. Written informed consent for participation was not required for this study in accordance with the national legislation and the institutional requirements.

## Author contributions

YY: guidance and supervision of whole experiments. YW and MW: participate in the whole experiment, data analysis, and manuscript writing. WL: participate in fermentation experiments. XW: participate in data analysis. WK: provide technical guidance. WH: provide experimental funding. JZ: provide experimental funding and supervise experiments. GX: guide the entire experiment and provide experimental raw materials. All authors contributed to the article and approved the submitted version.
